# Cooking heroin the Turkish way: chemical investigation on an unusual heroin preparation method

**DOI:** 10.1186/s12954-020-00457-1

**Published:** 2021-01-07

**Authors:** Georges Dahm, Claudia Allar, Raoul Schaaf, Adèle Bourmaud, Serge Schneider

**Affiliations:** 1 Laboratoire National de Santé, Service de toxicologie analytique - chimie pharmaceutique, 1, Rue Louis Rech, 3555 Dudelange, Luxembourg; 2 Abrigado, 8, route de Thionville, 2610 Luxembourg, Luxembourg

**Keywords:** Heroin purity, Standard cooking, “Turkish” cooking

## Abstract

**Background:**

Reports from experienced heroin users about an alternative and appreciated but harmful so-called “Turkish” heroin preparation technic led to the chemical investigation of the compounds produced during this process and investigation of the presence of other psychoactive contaminants.

**Methods:**

Comparison of diacetylmorphine, 6-monoacetylmorphine, morphine, paracetamol and caffeine concentrations were performed in the non-processed material, after processing according to the standard and to the alternative preparation methods using liquid chromatography coupled to quadrupole time of flight mass spectrometry followed by statistical evaluation of the results.

**Results:**

The two preparation methods had in common a diminution of diacetylmorphine as compared to the starting material but significantly more 6-monoacetylmorphine was produced using the “Turkish” preparation method as compared to the standard method.

**Conclusion:**

The high amount of psychoactive 6-monoacetylmorphine may have an impact on the reported effects of heroin using the “Turkish” preparation procedure.

## Introduction

**Fig. 1:**
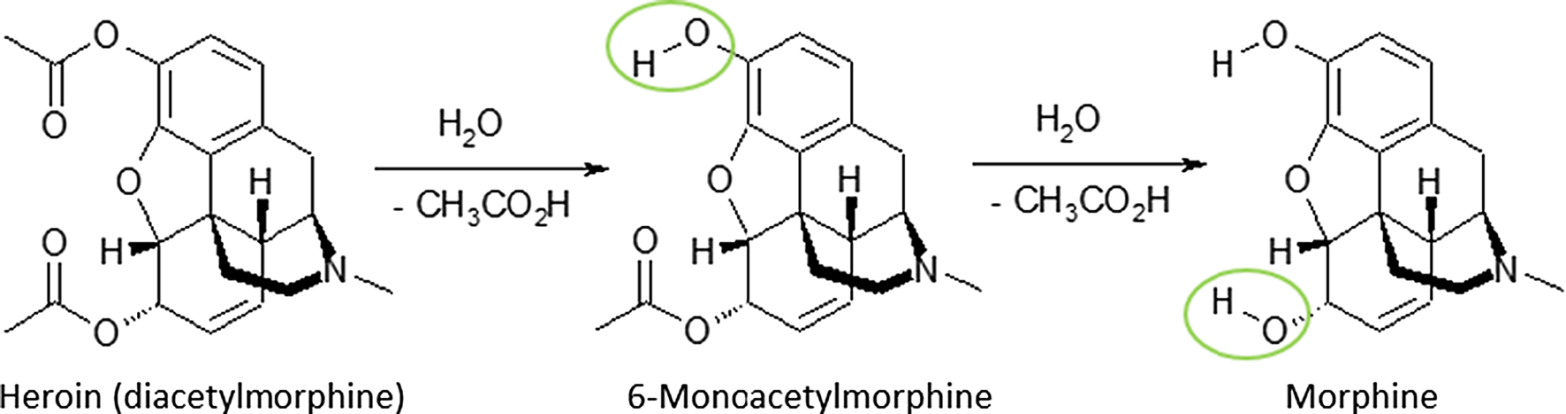
Degradation reaction of heroin to 6-monoacetylmorphine and morphine

In Western Europe, brown heroin (diacetylmorphine) is usually sold as the free base on the black market.^1^ The addition of acid, most frequently ascorbic acid or citric acid, is thus mandatory to obtain a water-soluble product usable for intravenous (IV) injection.^2^ Heroin and its degradation products 6-monoacetylmorphine (MAM) and morphine are responsible for the effect on the drug consumer (Fig. 1). This process is a metabolic pathway but can also occur during the preparation of the drug by heating.

While toxicological data and drug composition are closely monitored in most states in Europe and Northern America, the impact of heroin preparation methods is rarely investigated in the scientific literature and most information available is through consumer interviews.^3^^,^^4^ Thermal degradation, the breaking of chemical bounds by heat, has been reviewed in 1985 by Cook and Brine^5^ and by Bell and Nida in 2015.^6^

Most of the heroin preparation reports focus on the “standard” method, which consists in mixing the heroin powder with an acid, adding water or physiological saline solution and applying heat to dissolve the powder without notable color changes, resulting in a slight decrease of heroin and increase of its degradation products^2^. During interviews and discussions, at the drug consumption room “Abrigado” in Luxembourg City, about one-third of heroin consumers have reported the use of an alternative preparation method in many cases or even exclusively, the so-called “Turkish” method.

**Fig. 2:**
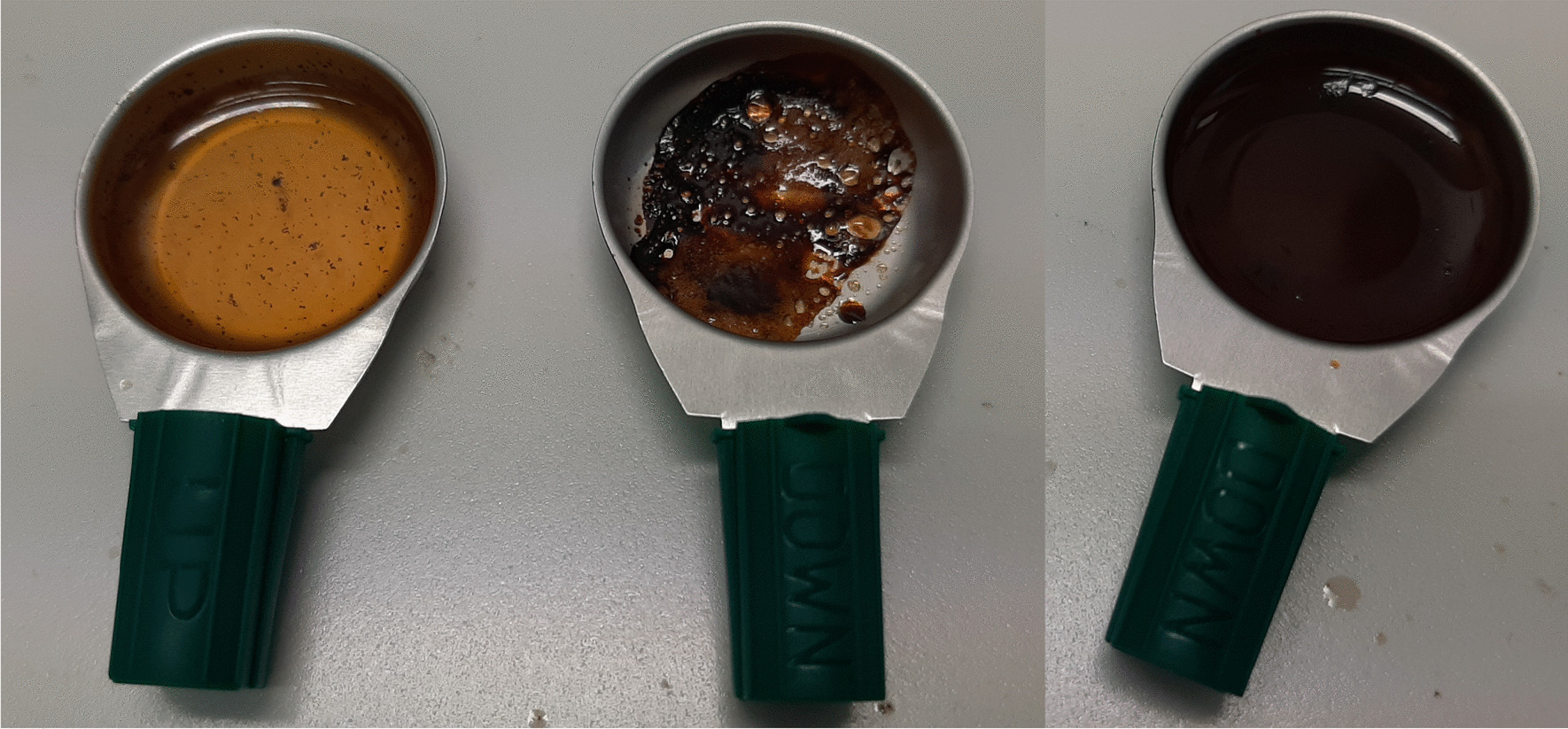
“Standard” preparation (left), “Turkish” preparation before (middle) and after (right) saline water addition

In contrast to the “standard” method, in the “Turkish” one no water or saline solution is added to the sample in a first step. The heroin powder with an acid is heated with a lighter until the first bubbles appear and turns dark brownish. Then, the saline solution is added and the mixture is briefly heated again for optimal dissolution (Fig. 2). Eventually, the solution is soaked up into the syringe through a cotton filter.

**Fig. 3:**
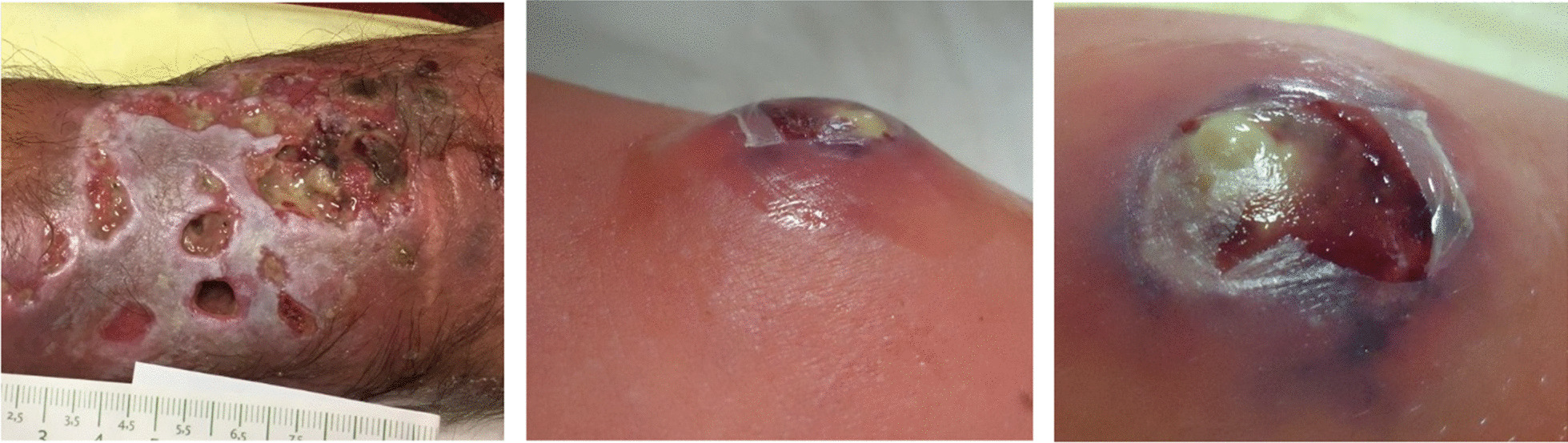
Abscesses observed at Abrigado due to “Turkish” preparation (Source: 7)

The “Turkish” preparation method is the method of many consumers. Interviews with health care personal with question regarding consumption habits and history, expectations of the products, price, smell and taste took place. It appears that consumers turn to the “Turkish” method to obtain more intense effects (“It pops better!”), better smell or taste (“Like coffee”), for the ritualized preparation and/or for the presumed elimination of contaminants and adulterants in the samples. However, compared to the “standard” preparation method, severe clinical side effects are often observed, i.e., abscesses and bad curable epidermal necrosis (Fig. 3).^7^ However, no increase in fatal or near fatal intoxication was reported. No information is available if the Turkish heroin use leads to a more frequent heroin consumption or needs a higher or lower dosage of the drug.

**Fig. 4:**
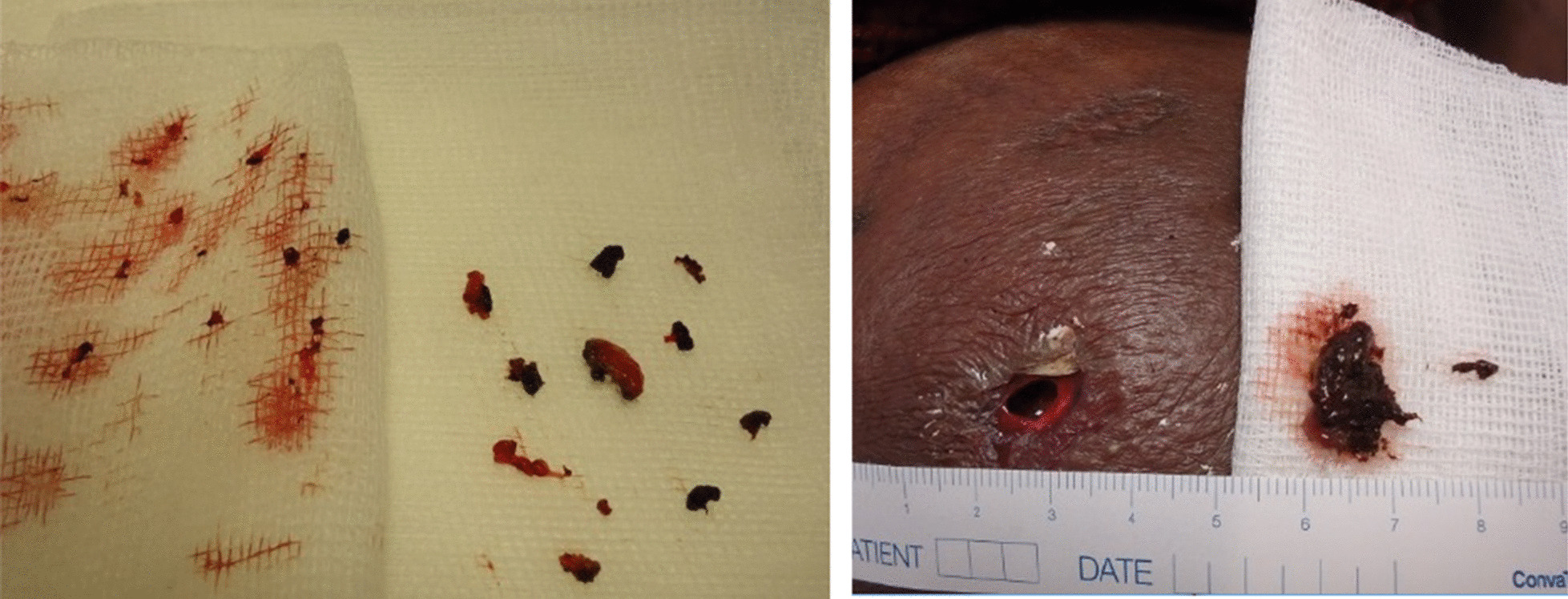
Syringe abscesses with collection of debris (Source: 7)

Extensively “Turkish” preparation produces soot particles (presumably due to partial carbonization in case of inhomogeneous heating of the powder), which are not completely removed even when using a cigarette or micro filter, or which clump again after being filtered. Due to the anesthetic effect of street diacetylmorphine, the consumer often does not notice whether and how much of the substance enters the tissue next to the vein leading to abscess formation. It is also noted that the epidermis covering the abscess necrotizes and dies off much faster. Subsequent wound treatment is difficult generally when these abscesses spontaneously open or must be treated surgically. The soot particles are firmly attached to the wound bed and their removal by enzymatic or mechanical wound cleansing remains difficult and tedious (Fig. 4).

Apart from the local consumer reports, brief mentions in a German newspaper in 2000^8^ and the 2015 evaluation report of a drug consumption room in Berlin,^9^ only anecdotal information about the “Turkish” preparation method is available on specialized internet forums^10^ or blogs.^11^ To our knowledge, no previous chemical investigation on this preparation method was performed.

In this paper, the main chemical degradation products produced when using the “Turkish” preparation procedure were investigated and compared to the initial sample composition and the standard preparation method.

The aims of the study were to understand the chemical processes occurring during the “Turkish” method and to explain the reported higher efficiency of the injected product. Furthermore, the results may give a scientific foundation for sensibilization of heroin users regarding health issues caused by the “Turkish” preparation method.

## Materials and methods

### Heroin samples

Brown heroin is the only form of heroin available at the Luxembourgish illegal drug market. A total of 30 independent samples received in 2019 from seizures of the Luxembourgish police or customs office have been randomly selected for analysis.

### Chemicals and materials

Diacetylmorphine (1 mg/mL, Lot# 687,046), 6-monoacetylmorphine (MAM, 1 mg/mL, Lot# 39,582), morphine (1 mg/mL, Lot# 88,283), paracetamol (1 mg/mL, Lot# 158,876) and caffeine (1 mg/mL, Lot# 980,098) were obtained from LGC Standards (Molsheim, France).

UPLC grade water with 0.1% formic acid (solvent A) and UPLC grade acetonitrile with 0.1% formic acid (solvent B) were purchased from Fisher Chemical (Waltham, MA, USA). Aluminum cookers (Apothicom Stericup, Chirana T. Injecta), ascorbic acid (Vitamin C, 100 mg, Chirana T. Injecta) and physiological saline solution (0.9% NaCl in water, Physio Flexo, 2 mL) were gifted from Abrigado (Luxembourg City).

### Heroin cooking

All samples were extensively homogenized before cooking.

The standard preparation method consisted in mixing 100 mg of heroin powder with 50 mg of ascorbic acid in the aluminum cup, prior the addition of 1 mL of saline solution. A gas lighter was used to heat uniformly the aluminum cup until complete dissolution of the solid material. This step took 15–60 s.

The “Turkish” preparation method consisted in mixing 100 mg of heroin powder with 50 mg of ascorbic acid in the aluminum cooker without addition of saline solution. After heating until liquefaction of the starting material (15–30 s), 1 mL of saline solution was added. A second heating step (30–60 s) was applied to obtain the final homogenous solution.

Independently of the preparation method, the final solutions were allowed to cool down to room temperature (> 2 min) before qualitative and quantitative analyses.

### Qualitative analysis

The analyses were performed using a LC-based high-resolution mass spectrometer in order to prevent the presence of artefacts that may result from the high temperature required in GC-based analyses (i.e., trans-acetylation).^12^

Screening for other compounds than diacetylmorphine, MAM, morphine, paracetamol and caffeine were performed on a G6550A ifunnel Q-ToF LC–MS system (Agilent, Waldbronn, Germany) equipped with a 1290 Infinity HPLC system. The system was operated using an Agilent MassHunter Workstation.

Chromatographic separation was achieved on a C18 column (Acquity UPLC BEH C18 Column, 130 Å, 1.7 µm, 1 mm × 50 mm). The elution was carried out by applying a mixture of solvents A and B. The compounds of interest were eluted using a linear gradient from 2 to 95% solvent B over 7 min at a flow rate of 0.3 mL/min and an oven temperature of 60 °C. The injection volume was 5 µL. The ESI interface was operated in positive ionization mode. The MS acquisition was performed in auto MS/MS mode (top 5) from 70 to 1700 m*/z* in MS and 50 to 1700 m*/z* in MS/MS. Acquisition rate was 3 spectra/s, and collision energy was determined using the following equation: 6 * *m/z*/100 + 4.^13^ Precursor selection was performed using an absolute threshold of 200 counts, a relative threshold of 0.01% active exclusion after 1 spectrum released after 0.15 min. Reference masses were 121.050873 and 922.009798 m*/z*. Identification was realized using exact *m/z*, retention time databases and MS/MS database (High Res NPS^14^). The corroboration of compounds identity was performed with mass tolerance of ± 0.005 Da and ± 0.05 Da for precursor and fragment ions, respectively, and retention times at ± 0.5 min, if known.

### Quantitative analysis

The same analytical system as for the qualitative screening was used for quantitative analyses. The compounds were eluted using a gradient from 0 to 100% solvent B over 26 min. The flow rate was 0.3 mL/min at 40 °C. The test solutions were diluted 100 times in 1000 µL of a mixture of solvent A and solvent B (9/1 V/V); 5 µL were injected into the LC-Q-ToF system. The MS acquisition was realized in positive MS mode from 50 to 500 m*/z* with an acquisition rate of 3 spectra/s and no collision energy was applied. Reference masses were the same as for the qualitative analysis. The area under the curve (AUC) was used to quantify each compound of interest. The quantification was performed using a four-point external calibration curve (0, 0.05, 0.10 and 0.50 mg/L).

### Statistical evaluation

In order to access the variability of the “Turkish” cooking method, a repeatability test was carried out by analyses of 12 aliquots of the same heroin sample. The relative standard deviation (RSD) was calculated, for each compound of interest, using the following formula:

$${\text{RSD}} = \frac{s}{x} \times 100\,\left( \% \right)$$

with *s*: standard deviation and *x*: mean concentration.

To evaluate the concentration changes of a compound of interest depending on the cooking methods, the P-value of the two-tailed t-test was calculated. The significance level was set to 0.05 (*p* > 0.05: no significant difference).

## Results

Qualitative and quantitative analyses of the 30 heroin samples were carried out on the raw material (prior to any cooking step), and after going through both the standard and the “Turkish” cooking methods.

Prior to cooking, qualitative screening was performed on all samples in order to access the sample compositions. All samples contained diacetylmorphine, MAM, paracetamol and caffeine. Morphine was detected in five out of the 30 samples. No psychoactive substances other than diacetylmorphine, MAM and morphine were detected (i.e., cocaine, fentanyls, new psychoactive substances, etc.) and no adulterants other than paracetamol and caffeine either (i.e., grisoefulvine and strychnine). Furthermore, none of the diacetylmorphine degradation products described by Cook and Brine were detected in the samples prior or after any type of cooking^5^.

The compounds of interest were quantified in the samples prior to cooking. The results (Table 1) are in concordance with data published by the EMCDDA.^15^ In particular, diacetylmorphine concentration range was from 3.5 to 46.5%, mean concentration was 18.9% and median concentration was 20.3%. MAM and morphine concentrations were low, often < LOQ.

**Table 1:** Diacetylmorphine, MAM, morphine, total opiates, paracetamol and caffeine concentration in untreated samples

Before cooking (%)	Mean	Median	Min	Max
Diacetylmorphine	18.9	20.3	3.5	46.5
MAM	2.8	2.3	0.19	10.7
Morphine	0.1	< LOQ	< LOQ	1.7
Total opiates	21.9	22.7	5.2	58.9
Paracetamol	29.5	28.1	0.5	56.0
Caffeine	14.2	14.1	0.6	26.0

Applying the standard cooking procedure overall mean concentrations of diacetylmorphine, MAM, morphine and adulterants did not change significantly (two-tailed *p* > 0.05). All changes for opiates were < 2.0%; changes for paracetamol and caffeine were slightly higher (i.e., up to -5.3% for paracetamol at high concentration), but not significant (*p* > 0.05). Morphine was detected in all samples but most of them below the limit of quantification (LOQ: 0.1%). All results are summarized in Table 2.

**Table 2:** Concentrations of opiates and adulterants in the heroin samples after the standard preparation method. Numbers in brackets are changes compared to untreated samples

Standard cooking (%)	Mean	Median	Min	Max
Diacetylmorphine	18.0 (− 0.9)	19.3 (− 0.9)	3.6 (+ 0.1)	44.7 (− 1.8)
MAM	2.9 (± 0)	2.8 (+ 0.5)	0.3 (+ 0.2)	11.6 (+ 0.9)
Morphine	0.1 (± 0)	< LOQ (± 0)	< LOQ (± 0)	1.9 (+ 0.2)
Total opiates	21.0 (− 0.9)	22.1 (− 0.7)	4.4 (− 0.8)	58.2 (− 0.6)
Paracetamol	28.0 (− 1.5)	26.3 (− 1.8)	0.1 (− 0.5)	50.7 (− 5.3)
Caffeine	13.8 (− 0.4)	13.5 (− 0.6)	0.3 (− 0.2)	26.6 (+ 0.5)

Applying the “Turkish” preparation method led to larger amount of diacetylmorphine being deacetylated into MAM (mean + 8.8% compared to untreated samples) and, to a lesser extent, to morphine (mean + 1.1%). Morphine was > LOQ in all samples but mean and median concentrations remained low compared to diacetylmorphine and MAM. Overall, the total opiates concentration diminished only 4.1% compared to untreated samples.

Changes for paracetamol and caffeine were higher than for the standard cooking method but remained well below changes observed for diacetylmorphine. All results are summarized in Table 3.

**Table 3:** Concentrations of opiates and adulterants in the heroin samples after the “Turkish” preparation method. Numbers in brackets are changes compared to untreated samples

“Turkish” cooking (%)	Mean	Median	Min	Max
Diacetylmorphine	5.0 (− 13.9)	4.6 (− 15.7)	0.2 (− 3.3)	15.8 (− 30.7)
MAM	11.6 (+ 8.8)	12.5 (+ 10.3)	0.8 (+ 0.6)	34.5 (+ 23.8)
Morphine	1.2 (+ 1.1)	0.6 (+ 0.6)	0.2 (+ 0.2)	4.9 (+ 3.3)
Total opiates	17.8 (− 4.1)	19.7 (− 3.1)	2.7 (− 2.5)	55.2 (− 3.7)
Paracetamol	25.9 (− 3.6)	24.5 (− 3.7)	0.2 (− 0.4)	49.6 (− 6.4)
Caffeine	12.8 (− 1.4)	13.0 (− 1.1)	0.5 (− 0.1)	22.9 (− 3.1)

The variability of the “Turkish” cooking method was assessed through a repeatability test performed on one sample containing 27.9% of diacetylmorphine. Twelve aliquots underwent the same cooking procedure under repeatability conditions. The results indicate that the “Turkish” method is highly repeatable (RSD 9.5% for MAM formation and 10.6% for total opiates variation). All results are summarized in Table 4.

**Table 4:** Repeatability test of “Turkish” cooking method

(%)	Diacetyl morphine	MAM	Morphine	Total opiates	Paracetamol	Caffeine
Mean	2.3	23.5	0.7	26.5	26.3	16.3
SD	2.3	2.2	0.3	2.8	2.0	1.7
RSD	99.8	9.5	37.9	10.6	7.5	10.3

## Discussion

The two cooking methods did not significantly (*p* > 0.05) change the combined concentrations of diacetylmorphine, MAM and morphine in the final solutions. Also, the concentration of paracetamol and caffeine did not changed significantly using both cooking methods (*p* > 0.05) rejecting the burning or disappearing of adulterants claimed by some consumers to take place when applying the “Turkish” cooking method.

**Fig. 5:**
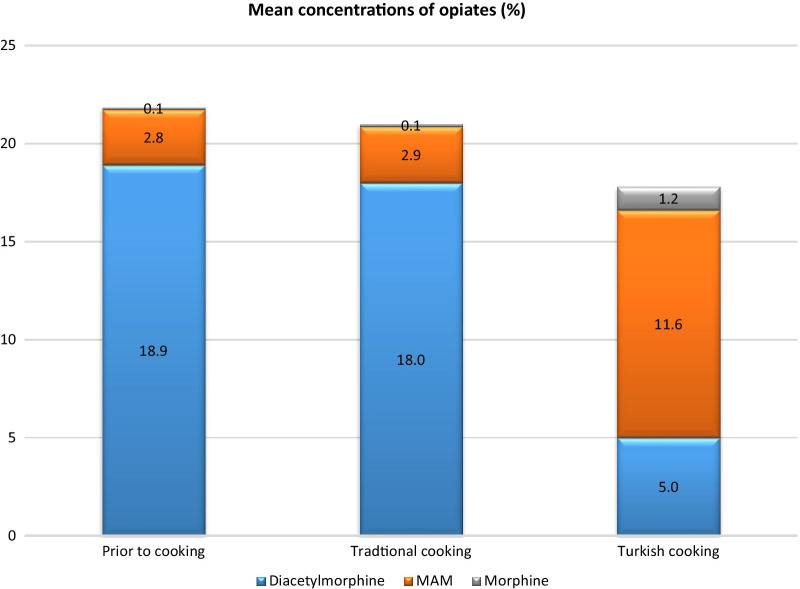
Mean concentrations of opiates in heroin powder prior to cooking, after standard and after “Turkish” cooking

The two tailed t-tests revealed no significant difference in MAM concentrations in initial heroin powder and the standard preparation (*p* > 0.05). Yet, a significant difference was observed when the “Turkish” preparation method is used (*p* < 0.05). The main finding in this study is that overall diacetylmorphine/MAM ratios changed when switching from the standard to the “Turkish” cooking method (Fig. 5). The diacetylmorphine/MAM ratio in untreated samples was 10.6, it remained roughly the same (11.2) after using the standard method but was inverted (0.66) when using the “Turkish” cooking method. In conclusion, “Turkish” cooking of heroin results in high conversion of diacetylmorphine to MAM.

The initial heroin, MAM, paracetamol and caffeine concentrations in the heroin powder did not influence the relative amount of MAM formed during standard or “Turkish” cooking. The correlation coefficient R^2^ was < 0.5 in all cases.

Applying heat to the aluminum cup when using the “Turkish” method, the temperature measured by a thermometer directly inside the powder raised to 120–180 °C, resulting in chemical degradation by breaking the weakest bond in diacetylmorphine, the ester group at position 3. In the presence of water, during the standard preparation method, the sample temperature will not exceed 100 °C, well below the diacetylmorphine degradation temperature, resulting in its dissolution without major formation of MAM.

A 4.1% decrease in overall opiate concentration was observed when using the “Turkish” method. However, it was not possible to identify the additional degradation products resulting from heroin heating. A possible toxic effect of the unknown compounds cannot be excluded.

It is well established that the blood–brain barrier (BBB) plays a central role in pain management when using opiates but also in the intense euphoric effects experienced after heroin injection. The high potency of diacetylmorphine, i.e., heroin, when compared to morphine has been attributed to its higher lipophilicity and the resulting better blood–brain permeability.^16^ Diacetylmorphine itself has only low affinity for the μ-opioid receptors^17^^,^^18^ it is a pro-drug and its effects are attributed to rapid formation in the blood^19^ and in the brain of the more potent receptor agonists MAM and morphine. Seleman and coworkers reported that MAM crosses the BBB not significantly slower than diacetylmorphine and 35 times faster than morphine.^20^ Pharmacokinetic studies showed that MAM blood concentrations reached maximum levels after 2–4 min only and MAM concentrations may be up to 18 times higher than those of diacetylmorphine.^21^ Consequently, it has been concluded that “MAM is likely the metabolite responsible for the acute effects of heroin”.^22^

The popularity of the “Turkish” preparation method among many heroin users is consistent with these results. As MAM is unstable and not readily available for use, the short heating of heroin powder results in the formation of significant amounts of MAM. When this preparation is diluted with saline solution and injected, MAM, not diacetylmorphine, is used and may be responsible for the perceived more intense effects of the heroin powder.

## Conclusion

Despite the claims of users of the “Turkish” cooking method, unsurprisingly, this process does not remove significant amounts of the major adulterants paracetamol and caffeine.

However, heating the heroin powder without the addition of water results in fast deacetylation of diacetylmorphine into MAM. This prevalence of MAM, the active psychoactive molecule, may be a trail of explanation for the reported intense effects of “Turkish” heroin when compared to the standard preparation method.

Using the “Turkish” preparation method conducts to more severe side effects, thus awareness of the consumers on the hazardous effects of this specific preparation method must be considered by health care workers.

## Data Availability

Not applicable.

## Abbreviations

MAM: 6-Monoacetylmorphine
IV: Intravenous
AUC: Area under the curve
BBB: Blood–brain barrier
RSD: Relative standard deviation

## Footnotes

1 European Monitoring Centre for Drugs and Drug Addiction: European Drug Report 2019: Trends and Developments. 2019. Publications Office of the European Union, Luxembourg. https://www.emcdda.europa.eu/publications/edr/trends-developments/2019_en. Accessed 25 Sept 2020.

2 Mørland J, Andersen J, Bogen I, Karinen R, Brochman G, Øiestad E, Vindenes V, Escolan F. Do they really inject heroin? A study on the possible changes in opioid content during heroin preparation for intravenous injection. Drug and Alcohol Dependence 2015; https://doi.org/10.1016/j.drugalcdep.2015.07.434.

3 Ponton R, Scott J. Injection preparation processes used by heroin and crack cocaine injectors. Journal of Substance Use. 2004;9:1.

4 Strang J, Keaney F, Butterworth G, Noble A, Best D. Different Forms of Heroin and their Relationship to Cook-Up Techniques: Data on, and Explanation of, Use of Lemon Juice and Other Acids. Substance Use & Misuse. 2001;36:5.

5 Cook CE, Brine DR. Pyrolysis products of heroin. J. Forensic Sci. 1985;30:1.

6 Bella S, Nida C. Pyrolysis of drugs of abuse: a comprehensive review. Drug Test Anal. 2015;7:6.

7 Koenen I, Allar C. Besonderheiten bei Drogenkonsumenten: Begleitende Krankheitsbilder. Internal Collaborator Formation Abrigado. 2020.

8 Taz.de. Aufkochen, aufziehen—und dann spritzen. 2000. https://taz.de/!1247023/. Accessed 25 Sept 2020.

9 Stöver H, Förster S, Hornig L, Theisen M. Evaluation der Nutzungsprofile der Drogenkonsumraumnutzer und -Nutzer und Nutzerinnen im Land Berlin. 2015.

10 Suchtmittel e.V. https://forum.suchtmittel.de/viewtopic.php?t=13303. Accessed 25 Sept 2020. Suchtmittel e.V. https://forum.suchtmittel.de/viewtopic.php?t=4157. Accessed 25 Sept 2020. Land der Träume B.V. https://www.land-der-traeume.de/forum.php?t=17366. Accessed 25 Sept 2020. Eve&Rave (Schweiz). https://www.eve-rave.ch/Forum/viewtopic.php?t=34986. Accessed 25 Sept 2020. Eve&Rave (Schweiz). https://www.eve-rave.ch/Forum/viewtopic.php?f=101&t=45504&p=2008024&hilit=t%C3%BCrkisch#p2008024. Accessed 25 Sept 2020. Eve&Rave (Schweiz). https://www.eve-rave.ch/Forum/search.php?keywords=tuerkisch&t=14468&sf=msgonly. Accessed 25 Sept 2020. Eve&Rave (Schweiz). https://www.eve-rave.ch/Forum/viewtopic.php?t=29029. Accessed 25 Sept 2020.

11 Berchner S. Kapitel 6: Ausgang Freiheit. 2019. https://berchnersblog.com/2019/01/13/kapitel-6-ausgang-freiheit/. Accessed 25 Sept 2020.

12 Dybowski R, Gough TA. A study of transacetylation between 3,6-diacetylmorphine and morphine. J. Chromatogr Sci. 1984;22:10.

13 Broecker S, Kuhlmann F, Wüst B, Zweigenbaum J. Toxicological Screening with the Agilent 6500 Series Accurate-Mass Q-TOF LC/MS and the Personal Compound Database and Library using the Broecker, Herre and Pragst Accurate Mass Spectral Library. Application Note Agilent Technologies, Inc. 2010.

14 Dalsgaard PW. HighResNPS. https://highresnps.forensic.ku.dk/Default.asp. Accessed 25 Sept 2020.

15 EMCDDA. Statistical Bulletin 2020 — price, purity and potency. 2020. https://www.emcdda.europa.eu/data/stats2020_en. Accessed 25 Sept 2020.

16 Chaves C, Remiao F, Cisternino S, Decleves X. Opioids and the Blood–Brain Barrier: A Dynamic Interaction with Consequences on Drug Disposition in Brain. Curr Neuropharmacol. 2017;15:8.

17 Hitchings A, Lonsdale D, Burrage D, Baker E. Top 100 drugs: clinical pharmacology and practical prescribing. 2014. ISBN 9,780,702,055,164.

18 Inturrisi CE, Schultz M, Shin S, Umans JG, Angel L, Simon EJ. Evidence from opiate binding studies that heroin acts through its metabolites. Life Sci. 1983;33:1.

19 Kamendulis LM, Brzezinski MR, Pindel EV, Bosron WF, Dean RA. Metabolism of cocaine and heroin is catalyzed by the same human liver carboxylesterases. J. Pharmacol. Exp. Ther. 1996;279:2.

20 Seleman M, Chapy H, Cisternino S, Courtin C, Smirnova M, Schlatter J, Chiadmi F, Scherrmann JM, Noble F, Marie- Claire, C. Impact of P-glycoprotein at the blood–brain barrier on the uptake of heroin and its main metabolites: behavioral effects and consequences on the transcriptional responses and reinforcing properties. Psychopharmacology (Berl.). 2014;231:16.

21 Gottås A, Øiestad EL, Boix F, Vindenes V, Ripel Å, Thaulow CH, Mørland J. Levels of heroin and its metabolites in blood and brain extracellular fluid after i.v. heroin administration to freely moving rats. British Journal of Pharmacology. 2013;170:3.

22 Chaves C, Remião F, Cisternino S, Declèves X. Opioids and the Blood–Brain Barrier: A Dynamic Interaction with Consequences on Drug Disposition in Brain. Current Neuropharmacology. 2017;15:8.

